# Pet, Pest, Profit: Patient! How Attitudes Toward Animals Among Veterinary Students in the Netherlands Differ According to Animal Categories and Student-Related Variables

**DOI:** 10.3390/ani15152222

**Published:** 2025-07-28

**Authors:** Angelika V. Dijkstra Klaasse, Monique R. E. Janssens, Daniela C. F. Salvatori

**Affiliations:** Anatomy and Physiology, Department Clinical Sciences, Faculty of Veterinary Medicine, Utrecht University, 3584 CL Utrecht, The Netherlandsd.salvatori@uu.nl (D.C.F.S.)

**Keywords:** animal welfare, attitude toward animals, veterinary students, empathy, animal categories, pet pest profit

## Abstract

Veterinarians are not just animal health professionals; they are also considered animal welfare experts. Animal-directed empathy, the ability to understand and match an animal’s emotional state, is essential for recognizing animal welfare issues. It is therefore a vital competency for veterinarians. The factors that play a role in shaping this empathy are animal, personal, and cultural influences, as well as the categorization of animals based on their benefit or harm to people: pet, pest or profit (used for economic purposes). We conducted a survey among veterinary students in the Netherlands to assess their levels of animal-directed empathy by scoring their attitude toward animals with the “Pet, Pest, Profit Scale”. Analysis of 321 completed surveys revealed that students showed the highest empathy for pets, the second-highest levels for pest animals, and the lowest levels for profit animals. Empathy levels also differed depending on career choice, background, and diet. These findings indicate that categorizing animals influences veterinary students’ empathy levels, which can lead to unrecognized welfare issues, especially for pest and profit animals. It is important to enhance empathy for these categories through targeted educational interventions to help prepare veterinary students for their responsibility as veterinarians, ensuring the welfare of all animals, whether pet, pest or profit.

## Simple Summary

Veterinarians are health professionals with a key role in protecting animal welfare. To ensure the well-being of animals, they need to be able to understand and connect with the animals’ emotional states. This animal-directed empathy can be influenced by animal categories—pet, pest, or profit (used for economic purposes)—by culture, by personal background and by animal traits. Our survey of veterinary students in the Netherlands showed they have the highest levels of empathy for pets, the second-highest levels for pest animals, and the lowest levels for profit animals. Empathy levels also vary according to factors such as career choice, student background, and diet. Less empathy can lead to welfare issues being overlooked, and it can affect decisions about treatment and euthanasia. Increasing animal-directed empathy in veterinary training, especially for pest and profit animals, can better prepare students for their responsibilities as veterinary professionals. This is essential to ensure the welfare of all types of animals—pet, pest, or profit.

## 1. Introduction

Animal welfare is becoming increasingly important to society, but even though most people would agree that we need to ensure the welfare of animals in human care and treat them respectfully, human concern is not equal for all animals [1,2,3,4]. The concept of animal welfare is much debated and constantly evolving [5,6].

We define animal welfare as the physical and mental well-being of sentient animals in relation to the conditions they experience throughout their life and at the time of their demise. An animal is deemed to have good welfare when it is in good health, comfortable, well-fed, secure, free from unpleasant conditions like pain, fear, and distress, and able to exhibit behaviors crucial for a predominantly positive physical and mental state and to react to changing circumstances. The maintenance of good animal welfare for animals kept by humans involves measures such as disease prevention, appropriate veterinary care, suitable shelter, effective management and nutrition, a stimulating and secure environment, and humane treatment and euthanasia. In animal welfare research, the focus has shifted from being free from negative experiences to having positive experiences or thriving, which includes expressing agency, having a sense of control, and being able to adapt to circumstances. All this makes “a life worth living” [7].

As animal health professionals, veterinarians are regarded by society as animal welfare experts and animal advocates [8]. They bear responsibility for the health and welfare of their patients. In the Netherlands, this responsibility is based on national legislation, the national and European Veterinary Code of Conduct, the veterinary oath, and the attainment targets of the Faculty of Veterinary Medicine of Utrecht University, the only veterinary faculty in the country [9,10,11,12]. For veterinarians to recognize welfare issues and to be motivated to prevent or resolve them, it is important that they have a deep understanding of what these welfare issues consist off and which emotions of the animal are involved under which circumstances. More specifically, the veterinarian needs the capacity for animal-directed empathy, the ability to experience another being’s point of view based on understanding (cognitively) or matching (affectively) their emotional state [13]. This makes animal-directed empathy [14] a core competency for veterinarians and veterinary students.

Empathy is a complex construct to measure, but the attitude of humans toward animals can be scored. Previous research has shown that empathy and attitude toward animals are significantly linked [15,16,17]. In addition, it has been identified that several factors can influence or correlate with humans’ attitudes toward animals. These factors can be animal-related, such as cuteness, likeness and sentience [18,19], personal, such as gender, diet, and experience with animals [20,21], or cultural, such as acceptance of animal use in traditional events like bullfighting [22]. A human’s attitude toward animals can also be affected by the category of animal one assigns the animal to [2,23].

Signal and Taylor suggest three main animal categories, based on their use and their benefit or harm to us: pet, pest, and profit animals. Pets are animals that are considered part of the family, pests are animals that harm us or damage human property, and profit animals are those used for monetary gains, such as production and research animals [23]. If you have a pet mouse, you will most likely place mice in the pet category; if you have been plagued by mice, you will consider them a pest category animal; and if you work as a researcher in a lab, you will probably see them as lab animals, which places them in the profit category. One species can thus be attributed to different categories, each with different sets of norms regarding their welfare status as a result.

In this way, categorization of animals influences what we as humans consider acceptable or not when dealing with them. It enables us to justify our attitude toward animals and even legalizes differences in the way they are treated. For example: the “in vivo ocular tolerance test” (Draize test, applying chemicals to rabbits’ eyes to assess the effect and damage) [24] is a legal procedure that can be performed in laboratory rabbits under specific circumstances but would be considered illegal and abuse when performed in a pet rabbit.

In turn, this categorization of animals is also shaped by various factors: not only by the features of the animal but also by a combination of the person’s cultural background and experiences. In many countries, consumption of dogs and cats is not acceptable, but eating cattle, pigs, and poultry is. The reason is that in those countries, dogs and cats are by culture categorized as pets, whereas cattle, pigs and poultry are categorized as food, i.e., profit animals.

When categorization influences veterinarians’ and veterinary students’ attitudes toward animals, it may significantly affect their animal-directed empathy, and as a result, the welfare of the animal involved. When veterinarians have lower levels of empathy toward certain animal categories, the welfare of the animals in those categories could be compromised. This could result in situations where welfare issues may not be readily recognized, prevented, or resolved, or the motivation to do so might be lacking. The potential influence of animal categorization is not limited to welfare issues, as it can also influence a vet’s decision about treatment or euthanasia [13,25,26,27].

This paper explores the relationship between the three mentioned animal categories and empathy with the Pet, Pest, Profit (PPP) Scale [23]. This scale, developed by Signal and Taylor, is based on and validated against the Animal Attitude Scale (AAS) developed by Herzog et al. [28]. Application of the AAS scale results in a single total score for animal-directed empathy and can be used to identify differences in empathy levels between people or over time when repeated with the same subject. However, this single score does not take into consideration different animal categories [23]. Therefore, a high score does not provide information about the relative levels of empathy a person has for different categories. To resolve this, Signal and Taylor developed the PPP scale, consisting of three subscales that can be used to measure attitudes toward animals in the pet, pest, and profit categories. Each subscale contains ten statements, resulting in a pet, a pest, and a profit score [23]. Using this method, differences in the attitudes of subjects toward animal categories in a certain human population can be identified. Since previous research has shown that empathy and attitude toward animals are significantly linked [15,16,17], measuring the attitude toward animals in different categories with the PPP scale is an indirect way of assessing empathy for animals in those categories.

This paper presents the results of a study that explored the potential differences in attitude toward animals in different categories, as a measure of animal-directed empathy. We did this by analyzing the pet, pest, and profit scores of veterinary students at Utrecht University. Based on the assumption that most students choose to study veterinary medicine because of their love for animals and their desire to help them, we wanted to find out whether students’ attitudes toward animals differ depending on the categorization of animals. We also explored whether the student-related variables year of study, career choice, background, diet, and gender were associated with the pet, pest, and profit scores of the veterinary students. Importantly, this study examines relative empathy across animal categories, not absolute levels of empathy. The PPP scale enables exploration of moral inconsistency by capturing how individuals assign differing levels of concern to animals based on their perceived social function.

The relationship between animal category and attitude toward animals has been previously studied in different populations, including veterinary practitioners and students [29,30,31,32,33]. One study investigating the effect of an animal welfare course on the attitude scores of students also included a small sample of veterinary students, but the main focus of that study was not the difference between categories but the effect of the welfare course on the scores [34]. These previous studies suggest that animal categories can influence the attitude toward animals and thus the level of animal-directed empathy among veterinary students and maybe also in the general population. In students, there was also an association with student-related factors, such as career choice, gender and diet [21,32,33,35].

Based on the outcomes of this previous research, we hypothesized that the total pet empathy score will be higher than the total pest and profit empathy scores. Regarding the student-related variables, we hypothesized that students in the early part of their studies, students with an ethical diet (vegetarian or plant-based), and female students would have higher pet, pest, and profit scores, whereas students choosing a career in production animals and students coming from a rural background would have lower pet, pest, and profit scores.

If these hypotheses are confirmed and animal-directed empathy depends on the position of an animal in one of the categories, this bias would be ethically problematic because we take as a starting point the belief that the interests of sentient animals should be considered equally or be respected. This means that we have moral duties toward them [36,37]. This study will focus on vertebrate species because they play a central role in veterinary practice and because there is overwhelming evidence that vertebrates are sentient beings [38,39].

## 2. Materials and Methods

### 2.1. Study Design

We used the Pet, Pest, and Profit Scale in an online survey on Qualtrics consisting of three parts (Supplementary File S1) to measure the PPP scores. The first part dealt with information and consent. The second part included eight questions on student demographics: gender, age, nationality, background, pet ownership, diet, study year, and career choice. The third part consisted of the “Pet, Pest, Profit Scale” [23].

The pet, pest and profit Likert subscales each contained ten Likert items (PPP statements) with the following scoring scheme: 1 = strongly disagree, 2 = disagree, 3 = neither agree nor disagree, 4 = agree, and 5 = strongly agree. Where applicable, the questions were reverse scored. This resulted in attitude scores for all three categories: the pet score, the pest score, and the profit score, ranging between ten and fifty each. A higher score indicates a more positive attitude toward animals, representing a higher level of animal-directed empathy. All the scores were converted into the percentage of the maximum possible (POMP) and analyzed as continuous data [40]. The POMP was used to facilitate easier comparison between the three scales by standardizing the scores to a common 0–100 range.

In order to use the Pet, Pest, Profit Scale in the Netherlands context, we made the following adjustments. The PPP statements were translated into the Dutch language, with back translation performed by a native English speaker. Instead of the pest example “cane toad” used in the original survey, we used “muskrat”, as cane toads are considered a pest in Australia but not in the Netherlands, where muskrats are considered a pest. Five veterinarians tested the survey for wording and flow. The data we wanted to collect was deemed anonymous by the GDPR officer of Utrecht University. The NVMO (Dutch Association for Medical Education) granted ethical approval for the study (ERB dossier 2022.1.8, 1 February 2022).

### 2.2. Participants and Settings

In agreement with the Directors of Education (Bachelor and Master) of the Utrecht Faculty of Veterinary Medicine, students were invited via the faculty’s Student Desk by email to participate in the research. The email contained an anonymous link to the survey and the NVMO-approved information letter to inform the students that participation was voluntary and anonymous, participation (or non-participation) would not affect their academic results, and their responses would only be used for scientific research.

The survey was sent out to 1390 students. The students received reminder emails 11 days and 32 days later, and the survey was taken offline after 50 days. The second reminder went to 1377 students, as 13 students had terminated their studies in the meantime. The survey was also promoted on social media (LinkedIn and Facebook), targeted at veterinary students and referring to the email they had received, without sharing the survey link itself on the internet.

To avoid potential response bias, the aim of this study was described as “gaining an understanding of the human–animal relationship in veterinary students” rather than assessing attitudes toward different animal categories [17].

### 2.3. Data Analysis

Based on the population size of 1390, an ideal sample size of 301 respondents was determined with a 95% confidence level and a 5% margin of error [41]. The raw data were exported from Qualtrics, checked, and cleaned (incomplete responses, duplicates, missing data, etc.). Incomplete surveys were excluded from the analysis. We analyzed the data in SPSS (version 28.0, IBM Corp., Armonk, NY, USA) with a significance level determined at *p* < 0.05.

Three measured variables were not analyzed. The question about student age was disqualified because it contained an unclear answer option. Secondly and thirdly, there was lack of contrast for the variables nationality and pet ownership. This was to be expected as most students attending the Dutch-language veterinary program at Utrecht University are Dutch, and due to the nature of the study, there are not many students who have never had a pet.

### 2.4. Outcomes of Interest

(1) We measured the internal consistency of the Pet, Pest, and Profit Scale for the current research population with Cronbach’s alpha.
(2) We tested the differences between the pet, pest, and profit scores with the Friedman test, a nonparametric alternative to the analysis of variance based on ranks, followed by a pairwise post hoc test with Bonferroni correction.
(3) We tested whether the pet, pest, and profit scores differed depending on student-related variables (year of study, career choice, background, diet, and gender) with a one-way analysis of variance or, in the case of violated equal variance, a Kruskal–Wallis test, followed by an appropriate pairwise post hoc test with Bonferroni correction.

## 3. Results

A total of 360 students responded to the survey. After removing incomplete surveys, possible bots, and duplicates, 321 responses were left to be included in the analysis. Respondents (Table 1) were evenly spread over the 6 study years. Years 1–3 cover the Bachelor phase and years 4–6 cover the Master phase. Respondents were mainly female (nearly 88%), which is in line with the high percentage of female veterinary students at the faculty. The students’ background was mainly village/town, followed by rural and urban. These categories were not based on population numbers but on self-reported personal experience. The largest career choice group was the group of veterinary students specializing in small animals (41%), followed by “large animal veterinarian” students and “equine animal veterinarian” students (both 19%). Most respondents did not follow a vegetarian or plant-based diet (70%), 26% partly followed a diet without meat, fish, dairy or eggs or a combination thereof, and 4% consumed plant-based products only. Most students were Dutch (99.4%), and almost everyone had had a pet (98.1%) at some point in their lives. The latter two results are not surprising given the nature of the veterinary course, which is offered in the Dutch language and attracts students with a relationship with animals.

### 3.1. Reliability of Pet, Pest, and Profit Subscales

The pet, pest, and profit subscales consisted of 10 Likert items each. Testing the subscale reliability for this population with Cronbach’s alpha resulted in the following: pet (α = 0.771), pest (α = 0.857), and profit (α = 0.846), with these α values considered acceptable [42].

### 3.2. Attitude Toward Animals Scores for Pet, Pest, and Profit Categories

**Figure 1:**
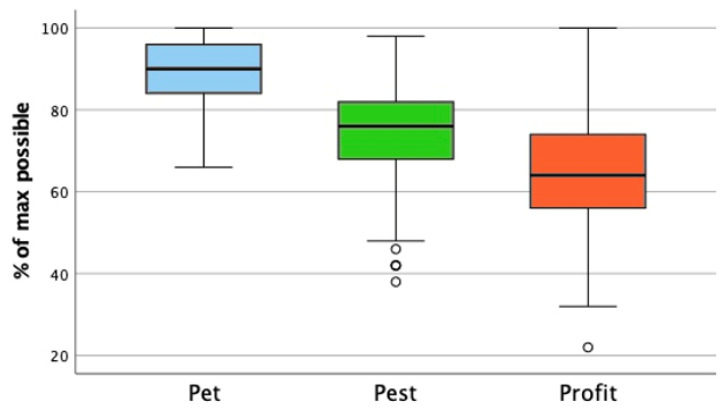
Attitude toward animals scores across categories. The pet score distribution was skewed left due to a ceiling effect also reported in other studies, so we compared the pet, pest, and profit scores with the Friedman test [34,43,44]. The *p*-value of <0.001 (χ^2^ = 489.571) indicated that there was a significant difference between at least two groups. Post hoc testing with Bonferroni correction for multiple comparisons between groups revealed statistically significant differences between the pet and pest, pet and profit and pest and profit scores (*p* < 0.001). This indicates that the attitude toward animals score differed across animal categories in this sample of veterinary students. Their attitude toward animals score is higher for animals they consider pets, followed, respectively, by animals they categorize as pests or profit animals.

The median score, as the percentage of the maximum possible, was highest for the category pet (90.0% [IQR = 12]), followed by pest (76.0% [IQR = 14]), with the lowest score for profit (64.0% [IQR = 18]) (Figure 1).

The boxplot illustrates the distribution of the attitude toward animals scores for the categories pet, pest, and profit, expressed as a percentage of the maximum possible score. The whiskers represent the minimum and maximum scores within the 1.5× interquartile range; the circles denote outliers beyond this range. The median score was highest for pet (90.0% [IQR = 12]), followed by pest (76.0% [IQR = 14]), and lowest for profit (64.0% [IQR = 18]). The pet category exhibited a left-skewed distribution due to a ceiling effect. The Friedman test indicated a significant difference between at least two groups (χ^2^ = 489.571, *p* < 0.001), and the post hoc Bonferroni comparisons confirmed the significant differences between all the category pairs (*p* < 0.001). These results suggest that veterinary students’ attitudes toward animals differ significantly depending on their categorization, with the most positive attitudes toward pets, followed by pests and profit animals.

### 3.3. Study Year

**Figure 2:**
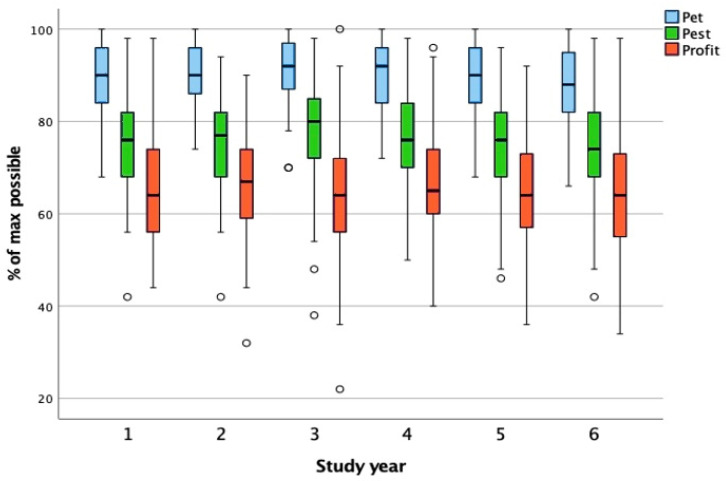
Comparison of pet, pest, and profit scores across study years. The boxplot presents the distribution of attitude toward animals scores for the categories pet, pest, and profit across six study years, expressed as a percentage of the maximum possible score. The whiskers represent the minimum and maximum scores within the 1.5× interquartile range; the circles denote outliers beyond this range. A one-way ANOVA revealed no statistically significant differences in the scores between study years for any category; therefore, no post hoc testing was conducted. These findings suggest that students’ attitudes toward animals remain relatively stable throughout their veterinary education.

The groups for this factor were first to sixth year. The one-way ANOVA did not result in statistically significant different pet, pest, and profit scores between the study year groups; therefore, no further post hoc testing was performed (Figure 2).

Test results: pet (F(5, 315) = 1.129, *p* = 0.300), pest (F(5, 315) = 1.352, *p* = 0.242) and profit (F(5, 315) = 0.621, *p* = 0.684).

### 3.4. Career Choice

**Figure 3:**
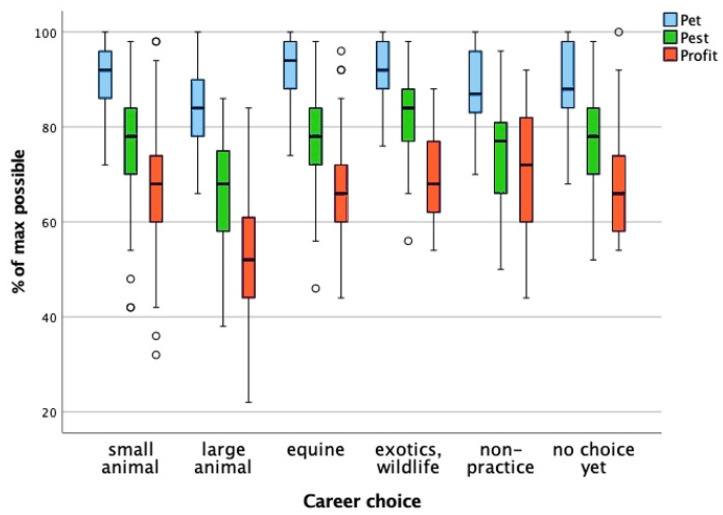
Comparison of pet, pest, and profit scores across career choice groups. The boxplot illustrates the distribution of attitude toward animals scores for the pet, pest, and profit categories across different career choice groups, expressed as a percentage of the maximum possible score. The whiskers represent the minimum and maximum scores within the 1.5× interquartile range; the circles denote outliers beyond this range. A one-way ANOVA indicated a significant effect of career choice on these scores (*p* < 0.001). Post hoc testing with Bonferroni correction revealed that students pursuing a career in large animal practice had significantly lower pet and pest scores compared to those choosing small animal, equine, or exotics/wildlife practice. Their profit scores were significantly lower than those of all other career groups. The effect sizes (partial eta-squared) were medium for pet (0.140) and pest (0.160), and high for profit (0.213), suggesting a strong association between career choice and attitudes toward animals categorized as profit animals. These findings indicate that students aiming for a career in large animal practice exhibit a markedly different attitude toward animals, particularly those considered profit animals.

The groups for this factor were as follows: small animal practice, large animal practice, equine practice, exotics/wildlife practice, non-practice (Netherlands Food and Consumer Product Safety Authority/research/education/business/government/NGO), and no choice made yet. The one-way ANOVA used to test the effect of career choice on the pet, pest, and profit scores resulted in a significant difference between at least two career groups (*p* < 0.001). Post hoc testing with Bonferroni correction revealed that the large animal practice group scores differed significantly from the other career choice groups (Figure 3). The students in this group had significantly lower pet and pest scores compared to the small animal, equine and exotics/wildlife groups. The lower profit score of these students was significant compared to all the other career choice groups.

The effect size (partial eta-squared) is a quantification of the variance in the pet, pest, and profit scores that can be explained by the career choice. The effect size was medium for pet (0.140) and pest (0.160) and high for profit (0.213). The high eta-squared for the profit score indicates a strong relation between career choice groups and profit score. This means that students choosing a career in large animal practice have significantly lower attitude toward animals scores, and this is most pronounced for animals categorized as profit animals.

### 3.5. Background of the Students

**Figure 4:**
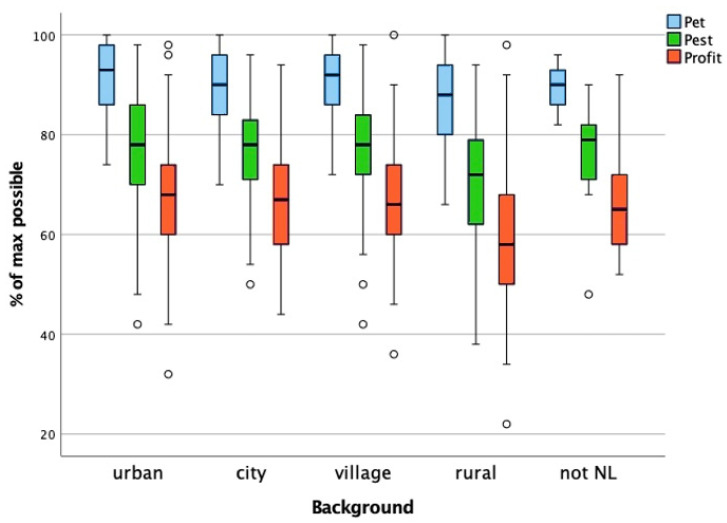
Comparison of the pet, pest, and profit scores across student backgrounds. The boxplot displays the distribution of attitude toward animals scores for the pet, pest, and profit categories across different student background groups, expressed as a percentage of the maximum possible score. The whiskers represent the minimum and maximum scores within the 1.5× interquartile range; the circles denote outliers beyond this range. A Kruskal–Wallis test was conducted for the pet scores due to a violation of the ANOVA equal variance assumption, revealing a statistically significant difference (H(4) = 23.900, *p* < 0.001). Post hoc testing with Bonferroni correction indicated that students from rural backgrounds had significantly lower pet scores than those from urban (*p* < 0.001) and village/town (*p* < 0.010) backgrounds. For the pest and profit scores, a one-way ANOVA indicated significant differences across background groups (pest: F(5, 315) = 11.957, *p* < 0.001; profit: F(5, 315) = 17.012, *p* < 0.001). Post hoc testing showed that students from rural backgrounds had significantly lower pest and profit scores compared to those from urban and village/town backgrounds (*p* < 0.001). The effect sizes (eta-squared) were medium for pet (0.078), pest (0.068), and profit (0.074), indicating that student background has a moderate impact on attitudes toward animals, with students from rural backgrounds consistently scoring lower across all three categories.

The groups for this factor were as follows: urban (NL Randstad), city (NL outside Randstad), village/town, rural, and not in the Netherlands. As the ANOVA equal variance assumption for the pet score was violated, we performed a Kruskal–Wallis test to compare the student background groups, which resulted in a statistically significant difference in the pet scores between at least two background groups (*p* < 0.001). Post hoc testing with Bonferroni correction showed that the students in the rural group had a statistically significant lower pet score than students in the urban (*p* < 0.001) and village/town group (*p* < 0.010) (Figure 4).

We tested the effect of student background on the pest and profit scores with a one-way ANOVA, which revealed a statistically significant difference in the pest and profit scores between at least two background groups. Post hoc testing with Bonferroni correction showed that the lower pest and profit scores of the students in the rural group were statistically significant compared to students in both the urban and the village/town group (*p* < 0.001), with a medium effect size (eta-squared) for pet (0.078), pest (0.068) and profit (0.074) (Figure 4). This means that students in this sample with a rural background have significantly lower pet, pest and profit scores than students with a village/town or urban background.

Kruskal–Wallis results: H(4)= 23.900, *p* < 0.001

Test results: pest (F(5, 315) = 11.957, *p* < 0.001) and profit (F(5, 315) = 17.012, *p* < 0.001)

### 3.6. Diet for Ethical Reasons

**Figure 5:**
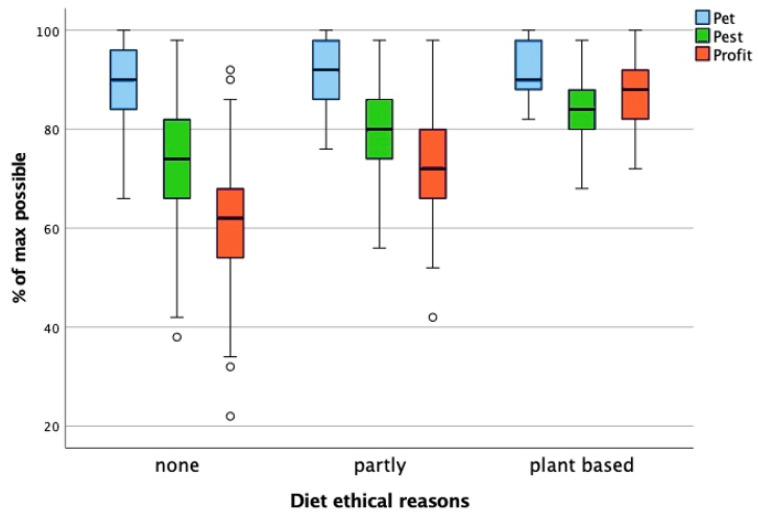
Comparison of the pet, pest, and profit scores across diet groups. The boxplot illustrates the distribution of attitude toward animals scores for the pet, pest, and profit categories among students following different diets for ethical reasons, expressed as a percentage of the maximum possible score. The whiskers represent the minimum and maximum scores within the 1.5× interquartile range; the circles denote outliers beyond this range. The one-way ANOVA revealed a statistically significant difference in the pet, pest, and profit scores between at least two diet groups (pet: F(2, 318) = 3.625, *p* = 0.028; pest: F(2, 318) = 12.451, *p* < 0.001; profit: F(2, 318) = 62.333, *p* < 0.001). Post hoc testing with Bonferroni correction revealed that students following a plant-based or partly ethical diet had significantly higher pest and profit scores than those in the no-diet group. Additionally, the partly ethical diet group had a significantly higher pet score than the no-diet group. The effect size (partial eta-squared) was low for pet (0.022), medium for pest (0.073), and very high for profit (0.282), indicating a strong association between diet choice and attitudes toward animals, particularly in the profit category. Students adhering to a diet for ethical reasons consistently demonstrated higher pest and profit scores compared to those who did not.

The groups for this factor were as follows: no diet (student does not follow a diet for ethical reasons), partly (diet without dairy, eggs/meat, fish), and plant-based diet. We use “partly” to describe diets that exclude meat but may include dairy, eggs, or fish. Our categories roughly align with unrestricted, vegetarian, and vegan diets. We performed a one-way ANOVA to test the effect of the diet groups on the pet, pest, and profit scores. This revealed a statistically significant difference in the pet, pest, and profit scores between at least two diet groups. Post hoc testing with Bonferroni correction showed that the plant-based diet and “partly diet” groups had statistically significant higher pest and profit scores than the no-diet group. Furthermore, the partly diet group also had a significantly higher pet score than the no-diet group (Figure 5). The effect size (partial eta-squared) was low for pet (0.022), medium for pest (0.073), and very high for profit (0.282). The students that follow a diet for ethical reasons had significantly higher pest and profit scores than students that did not. This was especially the case for the profit score.

Test results: pet (F(2, 318) = 3.625, *p* = 0.028), pest (F(2, 318) = 12.451, *p* < 0.001) and profit (F(2, 318) = 62.333, *p* < 0.001).

### 3.7. Gender

**Figure 6:**
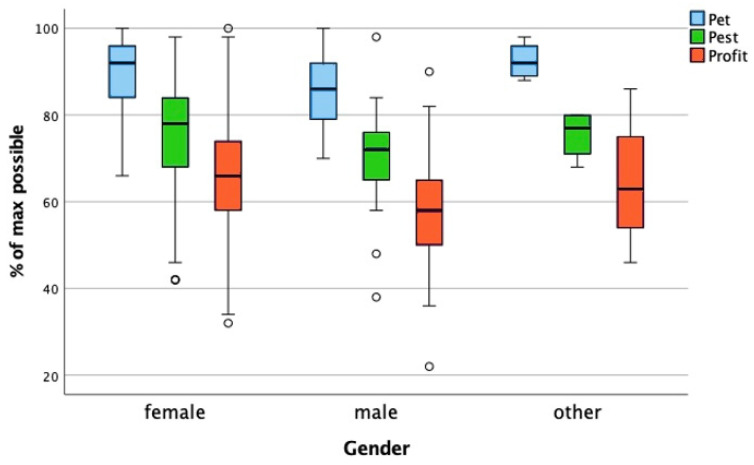
Comparison of the pet, pest, and profit scores across gender groups. The boxplot illustrates the distribution of attitude toward animals scores for the pet, pest, and profit categories among students with different gender identities, expressed as a percentage of the maximum possible score. The whiskers represent the minimum and maximum scores within the 1.5× interquartile range; the circles denote outliers beyond this range. A one-way ANOVA revealed a statistically significant difference in the pet, pest, and profit scores between at least two gender groups. Post hoc testing with Bonferroni correction showed that female students had significantly higher pet, pest, and profit scores than male students.

The groups for this factor were as follows: female, male, and other (neither, both, no answer). The one-way ANOVA performed to test the effect of gender on the pet, pest, and profit scores revealed that there was a statistically significant difference in the pet, pest, and profit scores between at least two gender groups. Post hoc testing with Bonferroni correction showed that the female group had significantly higher pet, pest, and profit scores than the male group (Figure 6). However, the effect size was small for all the categories; pet (0.033), pest (0.022), and profit (0.026).

Test results: pet (F(2, 318) = 5.404, *p* = 0.005), pest (F(2, 318) = 3.600, *p* = 0.028) and profit (F(2, 318) = 4.299, *p* = 0.014) scores.

The median score for the female subgroup (N = 282) was highest for category pet (92.0% [IQR = 12]), followed by pest (78.0% [IQR = 16]), with the lowest score for profit (66.0% [IQR = 16]).

The eta-squared quantifies the variance of the dependent variable, in this case the pet, pest and profit scores, that is explained by the independent variables, in this case the student-related variables and the animal categories. Note that in the context of the eta-squared, the term “independent variable” denotes a factor used to account for variance in the dependent variable, which represents the outcome being measured. The designation “independent” does not imply statistical or causal independence but rather reflects the variable’s role in the analytical model. A higher eta-squared value means that a larger part of the variance is explained by that specific variable, indicating a stronger relation. The percentage quantified by the eta-squared is the part of the differences in the pet score that can be explained by the student-related variables, while the remaining percentage is due to other factors that were not studied or random variation.

The pet score eta-squared of 27.3% is mainly explained by the variables career (14%) and background (7.8%). The pest score eta-squared of 32.3% is mainly explained by the variables career (16%), diet (7.3%), and background (6.8%). The profit score had the highest eta -squared of 59.5%, which is mainly explained by the variables diet (28.2%), career (21.3%), and background (7.4%).

Therefore, in this sample, diet, career, and background are the student-related variables that partly explain the variation in the pet, pest, and profit scores of the students, especially the variation in the profit score.

## 4. Discussion

This cross-sectional study, conducted in May 2022, explored the potential significant differences between the pet, pest, and profit scores among the veterinary student population at Utrecht University. We also studied the effect of student-related variables, study year, career choice, background, diet, and gender, on the pet, pest, and profit scores. We found statistically significant differences between the pet, pest, and profit scores: the pet score was higher than the pest and profit scores and the pest score was higher than the profit score. Other than expected, there was no significant effect of study year, but students with an ethical diet and female students did have significantly higher scores. Students choosing a career in production animals and students coming from a rural background scored significantly lower. These findings will be discussed in detail below. This implies that veterinary students’ attitudes toward animals correlate with animal categories and certain student-related variables. This bias in empathy among veterinary students might be problematic since it may result in the unequal treatment of animals in otherwise morally equal situations. Since this might also affect the assessment of and care for the welfare of animals, this bias needs to be addressed during the veterinary study program.

### 4.1. Pet, Pest, and Profit Scores

The statistically significant differences found between the pet, pest, and profit scores are in line with other studies [43,44], indicating that attitude toward animals among veterinary students correlates with the animal category. These results reflect differences in animal-directed empathy. Biases against pest and profit animals may lead to unrecognized welfare issues or diminished advocacy for these groups [13,25,26,27]. Profit animals are already subject to less stringent legal protection and may receive suboptimal care due to lower empathy levels [13]. Harmful procedures, e.g., traps for mice or rats (pest) and tail docking without anesthetic and pain relief in piglets (profit), are not deemed acceptable when applied to cats or dogs (pet) in the Netherlands. Therefore, classifying animals as pest or profit can be used to justify the way we treat them and allow for diminished concern for the welfare of these animals. Addressing these biases in veterinary medicine curricula is vital to equip students with the skills and motivation to uphold welfare standards across all animal categories.

### 4.2. Student-Related Variables

The finding that study year has no significant influence on the pet, pest, and profit scores in this study population was unexpected as other studies did find an effect. We expected a decline in empathy during the course of study, as found in other published studies [32,45]. A decline was found in Italian veterinary students’ Animal Attitude Score (AAS) over time [32], and Paul and Podberscek found that empathy toward animals decreased over time among male students [45]. The attitudes toward animal welfare in a study conducted among veterinary students in Malaysia fluctuated and indicated an improvement over time [46]. The authors did not have an explanation for this fluctuation, but they also found that the majority of the students had an anti-animal welfare attitude regardless of the year of study. This low baseline could explain the improvement over time. However, all these studies were cross-sectional, so no accurate conclusions can be drawn based on the change in attitude scores over the years. To investigate whether there is a significant trend and if this trend is positive or negative, a longitudinal cohort study surveying the students each year throughout their entire course of study is needed.

Career choice, background, and diet emerged as significant predictors of empathy levels. Finding lower attitude toward animals (ATA) scores in students with a preference for a career with large animals is consistent with results from other studies [21,44,47,48]. The exotic/wildlife group scored highest on the pest score, which seems logical as pest species are often wildlife species that are forced to compete with humans for resources. This was also found by Hazel et al. [44]. The significantly lower score for profit animals in the large animal practice group is worrisome as profit animals are already less protected by law, and their welfare is subservient to the benefit of humans. This is reflected in the Dutch Animals Act. Companion animals are legally protected against harm; however, enforcement is passive and complaint driven. Farm animals, on the other hand, are regularly inspected, but mainly for food safety compliance, and to a lesser degree, welfare. Legal exceptions permit practices in livestock that would be unacceptable in pets. This reflects a system where animals’ legal and moral protection depends more on their purpose (or category) than their capacity to suffer.

The lower pet, pest and particularly profit scores of the rural group are in line with studies that found reduced concern for animal welfare and rights in students from a rural background [21,48]. A study focused on Italian veterinary students found a less pro-animal welfare attitude, mainly in fifth-year students from the more urban north of the country. However, they also found a decline in their pro-animal welfare attitude over the years, which could offer an alternative explanation for this lower score in students with an urban background in this particular study year after more than four years of veterinary training [32]. Additionally, in this study, more than 60% of the students in the rural group have opted for a career in large animal practice, which is also the career choice group that scores lowest on the profit score; a lower profit score could thus be a cumulative effect of background and career choice. Veterinary faculties that want to address these biases and enhance empathy, especially for profit animals, could investigate mandatory targeted interventions, such as courses that highlight the sentience, communication, emotions, and welfare of farmed animals.

The relation between diet and attitude toward animals is also found in other studies [32,35,43,44,48]. Despite the fact that people generally care about animals, people tend to underestimate animals’ capacity to suffer when they are considered food, and moral concern is diminished [26]. The survey question did not specify animal welfare. The question was “Do you refrain from eating certain products for ethical reasons (not for health reasons or taste preferences)?” A follow-up question asking if the ethical reason was animal-welfare-related could have had added value because there are more reasons for a diet that avoids animal products, especially those based on the environment and climate change. However, people’s attitude toward environmental issues has also been identified as correlating with their attitude toward animals [49].

In line with other research [21,32,43,44], female students consistently scored higher on animal-directed empathy than males across all the categories, albeit with small effect sizes. Additionally, we performed a Friedman test to find out if the pet, pest, and profit scores were significantly different within the female subgroup. The result revealed that the differences between the pet and pest, pet and profit and pest and profit scores were statistically significant (*p* < 0.001). So, even though the female students had higher pet, pest, and profit scores than the male students, the animal category nevertheless had a statistically significant effect on the pet, pest, and profit scores.

This gender difference is widely reported in empathy research. It would be interesting to investigate whether this is due to societal norms, educational experiences, or intrinsic factors. And, though females score higher than males, the ATA score of female participants is predicted by category as well, with a significant difference between the pet (highest), pest, and profit (lowest) scores.

### 4.3. Implications

The variance in the animal-directed empathy scores between the pet, pest and profit categories and the relationship with the variables investigated in this study demonstrate that attitude toward animals is a construct that cannot be easily explained due to the multiple factors that play a role. However, the relevance of category, career choice and diet, particularly in terms of the pest and profit scores, is indicated by their significant effect sizes. The attitude toward animals categorized as profit animals is lowest in general and is linked to student-related variables, especially career choice and diet, and to a lesser degree, background. Therefore, differences in attitude toward animals and animal-directed empathy in veterinarians and veterinary students based on animal categories could have consequences for animals in their care as well as reducing advocacy for pest and especially for profit animals.

### 4.4. Recommendations

Keeping up with the latest research on animal welfare and animal mind, including sentience, cognition, feelings, and emotions of animals, is paramount for (future) veterinarians [25,43,44,50,51,52]. The significant differences in the pet, pest, and profit scores and the effect size of the variables in terms of these scores, especially for profit animals, emphasize the need to address the possibility of bias in empathy toward different categories of animals, in relation to these variables, during veterinary education [25,43,44,50,51,52]. This education could be provided in courses as part of the core curriculum, book club assignments, research projects on these topics, or even extra-mural work experience, e.g., in large animal rescue centers. The pet, pest, and profit scores can be measured pre- and post-course to assess the changes, noting that such designs indicate a correlation with the intervention rather than a causal relationship. This could be combined with research into which type of education is most effective, as previous studies report mixed results [43,44,52,53,54].

This study is the first of its kind in a sample population of veterinary students in the Netherlands. It would be worthwhile to follow up with a study exploring the attitude toward animals of educators at the university as their attitudes can also influence student attitudes [55]. Another sample population of interest is graduated veterinarians in the Netherlands; research in this group may provide more insight into how they perceive the veterinarian’s role in animal welfare and which variables influence their attitude toward animals and their animal-directed empathy. In general, we assume that more empathy leads to better treatment. However, there may come a point where empathy is so strong that it prevents wise decision-making in the best interests of the animal. With this article, we focus on the differences in empathy for different categories of animals, but this would also be an interesting point to investigate further.

## 5. Conclusions

This study shows that categorizing animals based on their benefit or harm to humans predicts and may affect the attitude toward animals (ATA) score in veterinary students, resulting in significantly different pet, pest, and profit scores. The student-related variables career choice, background, and diet significantly predict these pet, pest, and profit scores, with particularly negative implications for animals in the profit and pest categories. It is recommended to further study these and other influences on animal-directed empathy among veterinary students (such as experience with animals of different species and knowledge of animal sentience, mind, intelligence and emotions) to create awareness and help them prepare for their professional responsibility to optimize the welfare of all animal patients they are confronted with, regardless of the category we as humans assign them to.

### Limitations

This study has a number of limitations. The statements in the pet, pest, and profit subscales are quite different in nature, as noted by Van der Weijden [34]. The pet, pest, and profit subscales differ in tone and specificity, with the pet statements being more emotionally positive, the pest statements including specific behavioral scenarios, and the profit statements presenting utilitarian dilemmas. This reflects the authentic moral contexts in which people encounter these animal categories.

We acknowledge that the PPP adopts an oversimplification of complex relationships. In fact, the model reduces organisms to only three roles—pet, pest, or profit—which can oversimplify ecological and ethical realities. Many species fulfill multiple roles simultaneously or shift roles unpredictably due to ecological changes [56]. It could also be argued that the PPP scale suffers from an anthropocentric bias. The scale judges organisms based on their utility or nuisance to humans, rather than their ecological or intrinsic value [57]. In general, because ecosystems are dynamic, a model that promotes static categories may hinder adaptive pest or wildlife management [58].

While the PPP model has faced the reported criticism, its structured categorization remains a valuable tool for highlighting empathy discrepancies and moral inconsistencies in how animals are perceived and treated. The model’s utility lies in its ability to reveal implicit biases that shape veterinary and public attitudes. Additionally, the PPP model, validated against the Animal Attitude Scale (AAS) and psychometrically robust across cultural adaptations, enables targeted educational interventions to address empathy gaps [23]. The validation studies demonstrated strong internal consistency (Cronbach’s α > 0.85 for all subscales), a clear three-factor structure confirmed through factor analysis, and meaningful construct validity as shown by its correlation with the Animal Attitude Scale (AAS). A possible approach, using a combination of different scales to measure attitude toward animals, could have been of added value, but this would also have increased the risk of attrition.

In our study, the PPP subscales used a Likert scale consisting of ten 5-point Likert items. Although easy to analyze, some disadvantages could arise. Respondents can only choose from the given options, which may not represent their feelings accurately, as opposed to, for example, slider responses. Some respondents may avoid the most extreme options, while others may prefer extremes. The statements can also solicit socially acceptable answers. Likert items are considered ordinal data, but a Likert scale can be treated as continuous data as long as the items are related within the scale [34].

Another limitation is the fact that three measured variables could not be included in the analyses for the following reasons: the student age response options were not clear, and there was lack of contrast for the variables nationality and pet ownership. This was to be expected as most students at the Utrecht Faculty of Veterinary Medicine are Dutch, and due to the nature of the course of study, most students had lived with pets at some point in their lives.

The final limitation has an external cause: shortly after the invitation to the survey was sent to the students, the survey was unavailable for eight days due to a technical issue with the university account. This may have affected the number of respondents and caused some of the responses to be incomplete. Despite this mishap, the minimum sample size was acquired. Anyway, we argue that by contextualizing attitudes through the PPP framework, educators and policymakers can better understand and challenge the moral compartmentalization of animal categories, thereby strengthening advocacy and welfare standards across species.

## Figures and Tables

**Table 1:** Demographic factors of the veterinary student sample. This table provides an overview of key demographic characteristics of the surveyed veterinary students (N = 321). It includes information on the gender distribution, geographical background, dietary choices, study year, and intended career paths. These demographic factors characterize the sample and provide context for understanding the student population in this study.

Student Variable	Subgroups	N = 321 (100%)
**Gender**	Female	282 (87.9%)
	Male	35 (10.9%)
	Other (both 1, neither 1, no answer 2)	4 (1.2%)
**Background**	Urban	82 (25.5%)
	City	32 (10.0%)
	Village/town	104 (32.4%)
	Rural	95 (29.6%)
	Outside the Netherlands	8 (2.5%)
**Diet**	No	223 (69.5%)
	Partly (no dairy/eggs/meat/fish)	85 (26.48%)
	Yes (no products of animal origin)	13 (4.05%)
**Study year**	First year	57 (17.8%)
	Second year	60 (18.7%)
	Third year	55 (17.1%)
	Fourth year	46 (14.3%)
	Fifth year	48 (15.0%)
	Sixth year	55 (17.1%)
**Career choice**	Small animal practice	131 (40.8%)
	Large animal practice	60 (18.7%)
	Equine practice	61 (19.0%)
	Exotics/wildlife practice	28 (8.7%)
	Non-practice (food safety, research, education, industry, government NGO)	24 (7.5%)
	No choice made yet	17 (5.3%)

## Data Availability

Dataset available on request from the first author.

## Supplementary Materials

The following supporting information can be downloaded at: https://www.mdpi.com/article/10.3390/ani15152222/s1, Supplementary File S1: questionnaire.

## Abbreviations

AAS	Animal Attitude Scale
ANOVA	Analysis of Variance
ATA	Attitude Toward Animals
ERB	Ethical Review Board
GDPR	General Data Protection Regulation
IQR	Interquartile Range
KNMvD	Koninklijke Nederlandse Maatschappij voor Diergeneeskunde (Royal Dutch Veterinary Association)
NGO	Non-Governmental Organization
NL	Nederland (the Netherlands)
NVMO	Nederlandse Vereniging voor Medisch Onderwijs (Dutch Association for Medical Education)
PPP	Pet, Pest, and Profit (Scale)
SPSS	Statistical Package for the Social Sciences
UU	Utrecht University (Utrecht University)

## References

[1] Bradley A., Mennie N., Bibby P.A., Cassaday H.J. (2020). Some Animals Are More Equal than Others: Validation of a New Scale to Measure How Attitudes to Animals Depend on Species and Human Purpose of Use. PLoS ONE.

[2] Signal T., Taylor N., Maclean A.S. (2018). Pampered or Pariah: Does Animal Type Influence the Interaction between Animal Attitude and Empathy?. Psychol. Crime Law.

[3] Higgs M.J., Bipin S., Cassaday H.J. (2020). Man’s Best Friends: Attitudes towards the Use of Different Kinds of Animal Depend on Belief in Different Species’ Mental Capacities and Purpose of Use. R. Soc. Open Sci..

[4] Macauley L.P.J. (2018). Friends, Food, or “Free Egg Machines”? A Qualitative Study of Chicken Owners’ Perceptions of Chickens and Chicken Meat.

[5] Arndt S.S., Goerlich V.C., van der Staay F.J. (2022). A Dynamic Concept of Animal Welfare: The Role of Appetitive and Adverse Internal and External Factors and the Animal’s Ability to Adapt to Them. Front. Anim. Sci..

[6] Schukken Y.H., van Trijp J.C.M., van Alphen J.J.M., Hopster H. (2019). The State of the Animal in the Netherlands. Den Haag: Raad Voor Dierenaangelegenheden.

[7] Mellor D. (2016). Updating Animal Welfare Thinking: Moving beyond the “Five Freedoms” towards “A Life Worth Living”. Animals.

[8] Littlewood K.E., Beausoleil N.J. (2021). Two Domains to Five: Advancing Veterinary Duty of Care to Fulfil Public Expectations of Animal Welfare Expertise. Animals.

[9] Federation of Veterinarians of Europe (FVE) European Veterinary Code of Conduct. https://fve.org/cms/wp-content/uploads/FVE_Code_of_Conduct_2019_R1_WEB.pdf.

[10] Koninklijke Nederlandse Maatschappij voor Diergeneeskunde (KNMvD) Code Voor de Dierenarts. https://www.knmvd.nl/app/uploads/2022/06/Code-voor-de-Dierenarts-2022.pdf.

[11] Universiteit Utrecht Faculty of Veterinary Medicine Veterinaire Eed (Veterinary Oath). https://students.uu.nl/sites/default/files/dgk_eed_-_belofte_voor_de_dierenarts_mrt_2019.pdf.

[12] Universiteit Utrecht Faculty of Veterinary Medicine (2018). Eindtermen Masteropleiding Diergeneeskunde 2020.

[13] Norring M., Wikman I., Hokkanen A.-H., Kujala M.V., Hänninen L. (2014). Empathic Veterinarians Score Cattle Pain Higher. Vet. J..

[14] Prguda E., Neumann D.L. (2014). Inter-Human and Animal-Directed Empathy: A Test for Evolutionary Biases in Empathetic Responding. Behav. Process..

[15] Henry B.C. (2006). Empathy, Home Environment, and Attitudes toward Animals in Relation to Animal Abuse. Anthrozoös.

[16] Taylor N., Signal T.D. (2005). Empathy and Attitudes to Animals. Anthrozoös.

[17] Cuff B.M.P., Brown S.J., Taylor L., Howat D.J. (2016). Empathy: A Review of the Concept. Emot. Rev..

[18] Marriott S., Cassaday H.J. (2022). Attitudes to Animal Use of Named Species for Different Purposes: Effects of Speciesism, Individualising Morality, Likeability and Demographic Factors. Humanit. Soc. Sci. Commun..

[19] Batt S. (2009). Human Attitudes towards Animals in Relation to Species Similarity to Humans: A Multivariate Approach. Biosci. Horiz..

[20] Randler C., Adan A., Antofie M.-M., Arrona-Palacios A., Candido M., Pauw J.B.-D., Chandrakar P., Demirhan E., Detsis V., Di Milia L. (2021). Animal Welfare Attitudes: Effects of Gender and Diet in University Samples from 22 Countries. Animals.

[21] Serpell J.A. (2005). Factors Influencing Veterinary Students’ Career Choices and Attitudes to Animals. J. Vet. Med. Educ..

[22] Knight S., Vrij A., Bard K., Brandon D. (2009). Science versus Human Welfare? Understanding Attitudes toward Animal Use. J. Soc. Issues.

[23] Taylor N., Signal T.D. (2009). Pet, Pest, Profit: Isolating Differences in Attitudes towards the Treatment of Animals. Anthrozoös.

[24] Wilhelmus K.R. (2001). The Draize Eye Test. Surv. Ophthalmol..

[25] Hernandez E., Fawcett A., Brouwer E., Rau J., Turner P. (2018). Speaking Up: Veterinary Ethical Responsibilities and Animal Welfare Issues in Everyday Practice. Animals.

[26] Bratanova B., Loughnan S., Bastian B. (2011). The Effect of Categorization as Food on the Perceived Moral Standing of Animals. Appetite.

[27] Bubeck M.J. (2023). Justifying Euthanasia: A Qualitative Study of Veterinarians’ Ethical Boundary Work of “Good” Killing. Animals.

[28] Herzog H., Grayson S., McCord D. (2015). Brief Measures of the Animal Attitude Scale. Anthrozoös.

[29] Romero M.H., Escobar L., Sánchez J.A. (2022). Empathy Levels Among Veterinary Medicine Students in Colombia (South America). J. Vet. Med. Educ..

[30] Binngießer J., Wilhelm C., Randler C. (2013). Attitudes toward Animals among German Children and Adolescents. Anthrozoös.

[31] Morrison R., Maust-Mohl M., Charlton K. (2021). Friend, Foe, or Food: What Influences Students’ Attitudes Toward Animals?. Anthrozoös.

[32] Pirrone F., Mariti C., Gazzano A., Albertini M., Sighieri C., Diverio S. (2019). Attitudes toward Animals and Their Welfare among Italian Veterinary Students. Vet. Sci..

[33] Calderón-Amor J., Luna-Fernández D., Tadich T. (2017). Study of the Levels of Human–Human and Human–Animal Empathy in Veterinary Medical Students from Chile. J. Vet. Med. Educ..

[34] Weijden J.V.D. (2013). Attitudes towards the Use of Animals of Students Enrolled in Animal Welfare and Laboratory Science Courses in The Netherlands. Master Thesis.

[35] Menor-Campos D.J., Diverio S., Sánchez-Muñoz C., López-Rodríguez R., Gazzano A., Palandri L., Mariti C. (2019). Attitudes toward Animals of Students at Three European Veterinary Medicine Schools in Italy and Spain. Anthrozoös.

[36] Singer P. (2009). Animal Liberation.

[37] Korsgaard C.M. (2018). Fellow Creatures: Our Obligations to the Other Animals.

[38] Marino L. (2017). Thinking Chickens: A Review of Cognition, Emotion, and Behavior in the Domestic Chicken. Anim. Cogn..

[39] Dawkins M.S. (1980). Animal Suffering: The Science of Animal Welfare.

[40] Harpe S.E. (2015). How to Analyze Likert and Other Rating Scale Data. Curr. Pharm. Teach. Learn..

[41] Qualtrics Sample Size Calculator. https://www.qualtrics.com/blog/calculating-sample-size/.

[42] Guyatt G., Drummond R., Meade M.O., Cook D.J. (2015). Users’ Guides to the Medical Literature.

[43] Robbins J., Danielson J., Johnson A., Parsons R., Jorgensen M., Millman S. (2021). Attitudes towards Animals and Belief in Animal Mind among First-Year Veterinary Students before and after an Introductory Animal Welfare Course. Anim. Welf..

[44] Hazel S.J., Signal T.D., Taylor N. (2011). Can Teaching Veterinary and Animal-Science Students about Animal Welfare Affect Their Attitude toward Animals and Human-Related Empathy?. J. Vet. Med. Educ..

[45] Paul E.S., Podberscek A.L. (2000). Veterinary Education and Students’ Attitudes towards Animal Welfare. Vet. Rec..

[46] Azahar F.A.M., Fakri N.M.R.M., Pa M.N.M. (2014). Associations between Gender, Year of Study and Empathy Level With Attitudes towards Animal Welfare among Undergraduate Doctor of Veterinary Medicine Students in Universiti Putra Malaysia. Educ. Med. J..

[47] Cornish A.R., Caspar G.L., Collins T., Degeling C., Fawcett A., Fisher A.D., Freire R., Hazel S.J., Hood J., Johnson A.J. (2016). Career Preferences and Opinions on Animal Welfare and Ethics: A Survey of Veterinary Students in Australia and New Zealand. J. Vet. Med. Educ..

[48] Mariti C., Pirrone F., Albertini M., Gazzano A., Diverio S. (2018). Familiarity and Interest in Working with Livestock Decreases the Odds of Having Positive Attitudes towards Non-Human Animals and Their Welfare among Veterinary Students in Italy. Animals.

[49] Binngießer J., Randler C. (2015). Association of the Environmental Attitudes “Preservation” and “Utilization” with Pro-Animal Attitudes. Int. J. Environ. Sci. Educ..

[50] Knight S., Vrij A., Cherryman J., Nunkoosing K. (2004). Attitudes towards Animal Use and Belief in Animal Mind. Anthrozoös.

[51] de Waal F.B.M., Andrews K. (2022). The Question of Animal Emotions. Science.

[52] Hazel S., O’Dwyer L., Ryan T. (2015). “Chickens Are a Lot Smarter than I Originally Thought”: Changes in Student Attitudes to Chickens Following a Chicken Training Class. Animals.

[53] Ventura B.A., Terreaux C.M.H.A., Zhitnitskiy P.E. (2021). Veterinary Student Knowledge and Attitudes about Swine Change after Lectures and a Farm Visit. J. Vet. Med. Educ..

[54] Johnstone E.C.S., Frye M.A., Lord L.K., Baysinger A.K., Edwards-Callaway L.N. (2019). Knowledge and Opinions of Third Year Veterinary Students Relevant to Animal Welfare Before and After Implementation of a Core Welfare Course. Front. Vet. Sci..

[55] Tzioumis V., Freire R., Hood J., Johnson A.J., Lloyd J., Phillips C.J.C., McGreevy P.D. (2018). Educators’ Perspectives on Animal Welfare and Ethics in the Australian and New Zealand Veterinary Curricula. J. Vet. Med. Educ..

[56] Kopnina H. (2016). Wild Animals and Justice: The Case of the Dead Elephant in the Room. J. Int. Wildl. Law Policy.

[57] Crist E., Cafaro P., Crist E. (2012). Abundant Earth and the Population Question. Life on the Brink: Environmentalists Confront Overpopulation.

[58] Kremen C., Miles A. (2012). Ecosystem Services in Biologically Diversified versus Conventional Farming Systems: Benefits, Externalities, and Trade-Offs. Ecol. Soc..

