# Supplementary material for: Pet, Pest, Profit: Patient! How Attitudes Toward Animals Among Veterinary Students in the Netherlands Differ According to Animal Categories and Student-Related Variables
Source: Animals (Basel). 2025 Jul 28;15(15):2222. doi: 10.3390/ani15152222 (PMC12345569; doi:10.3390/ani15152222)
Supplement: Supplementary file 1 [file animals-15-02222-s001.zip › animals-3698257 - questionnaire.pdf]

Uitleg en consent

Welkom toekomstige collega, fijn dat je wilt bijdragen aan mijn onderzoek!

Door toestemming te geven ga je akkoord met het volgende:

- Ik heb de informatie in de email gelezen. Ik kon vragen stellen. Mijn vragen zijn voldoende beantwoord. Ik had genoeg tijd om over deelname te beslissen.
- Ik weet dat meedoen vrijwillig is en dat ik mijn toestemming kan intrekken op ieder moment van het onderzoek. Daarvoor hoef ik geen reden te geven.
- Ik weet dat als ik mij terugtrek, mijn gegevens tot dat moment gebruikt kunnen worden.
- Ik geef toestemming voor het verzamelen, bewaren en gebruiken van de door mij ingevoerde anonieme gegevens voor de beantwoording van de onderzoeksvraag in dit onderzoek.
- Ik geef toestemming voor hergebruik van de door mij ingevoerde anonieme gegevens na dit onderzoek voor nu nog onbekend onderzoek dat binnen het vakgebied van het (bio)medisch-onderwijs onderzoek valt. Hierbij worden de erkende ethische normen voor deze vorm van wetenschappelijk onderzoek in acht genomen.

Heb je hier vragen over? Mail mij dan s.v.p. op a.v.dijkstra@uu.nl

Toestemming

- ○ Ja, ik geef toestemming.
- ○ Nee, ik geef geen toestemming.

Student-gebonden informatie

Met welk geslacht identificeer je jezelf?

- ○ Vrouw
- ○ Man
- ○ Beide
- ○ Geen van beide
- ○ Zeg ik liever niet

Mijn leeftijd is

- Jonger dan 20 jaar
- 21 - 23 jaar
- 24 - 26 jaar
- 27 - 29 jaar
- 30 - 32 jaar
- 33 - 35 jaar
- 36 - 38 jaar
- ouder dan 39 jaar
- Zeg ik liever niet

Mijn nationaliteit is

- Nederlands
- Anders

Ik ben (grotendeels) opgegroeid
(selecteer s.v.p. wat het meest van toepassing is)

- in de randstad
- in een grote stad buiten de randstad
- in een groot dorp/kleine stad
- op het platteland
- buiten Nederland

Heb je een huisdier?

- Ja
- Niet op dit moment, maar in het verleden wel gehad
- Nee, ik heb nooit een huisdier gehad

Zijn er producten die je niet eet om ethische redenen? (dus niet vanwege gezondheid of smaak voorkeur)

- Ik eet geen vlees/vis
- Ik eet geen zuivel/eieren
- Ik eet geen vlees/vis en geen zuivel/eieren
- Ik eet geen enkel product van dierlijke oorsprong
- Nee, bovenstaande is niet van toepassing op mij

In het huidige academische jaar (2021-2022) ben ik

- ○ 1e-jaars student (Bachelor Diergeneeskunde)
- ○ 2e-jaars student (Bachelor Diergeneeskunde)
- ○ 3e-jaars student (Bachelor Diergeneeskunde)
- ○ 4e-jaars student (Master Diergeneeskunde)
- ○ 5e-jaars student (Master Diergeneeskunde)
- ○ 6e-jaars student (Master Diergeneeskunde)

Mijn **1e voorkeur** voor mijn toekomstige werkgebied is
(indien gemengde praktijk kies de optie van 1e voorkeur voor diersoort s.v.p.)

- ○ Praktijk Gezelschapsdieren
- ○ Praktijk Landbouwhuisdieren
- ○ Praktijk Paard
- ○ Praktijk Bijzondere dieren / Wildlife
- ○ Veterinaire volksgezondheid (NVWA)
- ○ Onderzoek
- ○ Onderwijs
- ○ Bedrijfsleven of Overheid
- ○ Not for Profit Dierenorganisatie
- ○ Geen van bovenstaande opties / weet ik nog niet

Pets-Pest-Profit vragen

Nu volgen **3 blokken met elk 10 stellingen** over respectievelijk huisdieren, plaagdieren en dieren voor economische doeleinden.

Met de pijlen links en rechts van de stelling kun je navigeren tussen de **stellingen** in een blok.
Wil je navigeren tussen de **vragen**, klik dan op de pijlen onderaan de pagina. Als deze niet zichtbaar zijn, scroll dan iets verder naar beneden.

HUISDIEREN

In welke mate ben je het eens of oneens met de volgende stellingen over huisdieren.

Kies s.v.p. de optie die het meest overeenkomt met jouw mening over **stelling 1 t/m 10:**

	Helemaal niet mee eens	Niet mee eens	Niet mee eens & niet mee oneens	Mee eens	Helemaal mee eens
**Stelling 1:** Zieke huisdieren hebben recht op diergeneeskundige zorg	○	○	○	○	○
**Stelling 2:** Huisdieren hebben recht op een leven vrij van angst en stress	○	○	○	○	○
**Stelling 3:** Ik beschouw mijn huisdier als een lid van mijn familie	○	○	○	○	○
**Stelling 4:** Mijn huisdier is een bron van troost (of zou dat zijn als ik een huisdier had)	○	○	○	○	○
**Stelling 5:** Alle huisdieren hebben elke dag aandacht nodig	○	○	○	○	○
**Stelling 6:** De gezondheid van huisdieren moet regelmatig gecheckt worden	○	○	○	○	○
**Stelling 7:** Huisdieren hebben recht op vers water en een passend dieet om gezond te blijven	○	○	○	○	○
**Stelling 8:** Ik word ongerust als mijn huisdier niet eet	○	○	○	○	○
**Stelling 9:** Huisdieren moeten op een geschikte manier gehouden worden met beschutting en een comfortable plaats om te rusten	○	○	○	○	○
**Stelling 10:** Huisdieren hebben recht op een leven vrij van pijn, verwondingen of ziekte	○	○	○	○	○

PLAAGDIEREN

In welke mate ben je het eens of oneens met de volgende stellingen over plaagdieren.

Kies s.v.p. de optie die het meest overeenkomt met jouw mening over **stelling 1 t/m 10:**

	Helemaal niet mee eens	Niet mee eens	Niet mee eens & niet mee oneens	Mee eens	Helemaal mee eens
**Stelling 1:** Plaagdieren hebben recht op vers water en een passend dieet om gezond te blijven	○	○	○	○	○

	Helemaal niet mee eens	Niet mee eens	Niet mee eens & niet mee oneens	Mee eens	Helemaal mee eens
**Stelling 2:** Hoewel we plaagdieren die problemen veroorzaken moeten aanpakken (b.v. muskusratten) moeten humane methodes gezocht worden	○	○	○	○	○
**Stelling 3:** Plaagdieren hebben recht op een leven vrij van ongemak	○	○	○	○	○
**Stelling 4:** Plaagdieren hebben recht op een leven vrij van angst en stress	○	○	○	○	○
**Stelling 5:** Plaagdieren hebben recht op het uiten van natuurlijk gedrag	○	○	○	○	○
**Stelling 6:** Plaagdieren hebben recht op een leven vrij van pijn, verwondingen of ziekte	○	○	○	○	○
**Stelling 7:** Het doden van plaagdieren die schade veroorzaken aan bezittingen van mensen is noodzakelijk	○	○	○	○	○
**Stelling 8:** Het is acceptabel om zelf plaagdieren te doden op een manier waar ik de voorkeur aan geef	○	○	○	○	○
**Stelling 9:** Het is acceptabel om zelf verwilderde dieren te doden op een manier waar ik de voorkeur aan geef	○	○	○	○	○
**Stelling 10:** Plaagdieren hebben geen waarde en moeten op elke mogelijke manier verwijderd worden	○	○	○	○	○

DIEREN VOOR ECONOMISCHE DOELEINDEN

In welke mate ben je het eens of oneens met de volgende stellingen over dieren voor economische doeleinden.

Kies s.v.p. de optie die het meest overeenkomt met jouw mening over **stelling 1 t/m 10:**

	Helemaal niet mee eens	Niet mee eens	Niet mee eens & niet mee oneens	Mee eens	Helemaal mee eens
**Stelling 1:** Mensen hebben het recht om dieren voor voedsel te gebruiken	○	○	○	○	○
**Stelling 2:** Transporteren van levende dieren is een acceptabele manier om geld mee te verdienen	○	○	○	○	○
**Stelling 3:** Het is acceptabel om dieren voor menselijk gewin te gebruiken	○	○	○	○	○
**Stelling 4:** Jagen voor geld is immoreel en moet stoppen	○	○	○	○	○
**Stelling 5:** Het is wreed om vogels in kooien te houden enkel voor massaproductie van eieren	○	○	○	○	○

	Helemaal niet mee eens	Niet mee eens	Niet mee eens & niet mee oneens	Mee eens	Helemaal mee eens
**Stelling 6:** Het is oké om dieren te gebruiken voor het testen van producten als cosmetica en schoonmaakmiddelen	○	○	○	○	○
**Stelling 7:** Intensieve veehouderij is noodzakelijk in de wereld van vandaag	○	○	○	○	○
**Stelling 8:** Transport van levende dieren zou verboden moeten worden	○	○	○	○	○
**Stelling 9:** De regelgeving rondom humaan doden en slachthuizen is niet streng genoeg	○	○	○	○	○
**Stelling 10:** Ik zou meer geld over hebben voor "diervriendelijke" producten (b.v. eieren; vlees)	○	○	○	○	○

Powered by Qualtrics
